# Evaluation of left ventricular flow field changes after stress in patients with nonobstructive coronary artery disease using ultrasonic flow vector imaging

**DOI:** 10.3389/fcvm.2024.1340289

**Published:** 2024-03-21

**Authors:** Dongmei Li, Xin Zhao, Qiuyu Xiao, Rui Yang, Zizhuo Li, Yuanyuan Xie, Xinyue Mao, Xi Li, Wenhan Hu, Yan Deng

**Affiliations:** ^1^ School of Medicine, University of Electronic Science and Technology, Chengdu, China; ^2^ Department of Ultrasound Medicine, School of Medicine, Chengdu Second People’s Hospital, Chengdu, China; ^3^ School of Medicine, Chengdu Medical College, Chengdu, China; ^4^ School of Medicine, North Sichuan Medical College, Nanchong, China; ^5^ Department of Cardiovascular Ultrasound and Cardiac Function, Affiliated Hospital of University of Electronic Science and Technology, Sichuan Provincial People’s Hospital Sichuan Provincial Key Laboratory of Ultrasonic Cardiac Electrophysiology and Biomechanics Sichuan Clinical Medical Research Center for Cardiovascular Disease National Clinical Medical Research Center for Cardiovascular Diseases Branch Center, Chengdu, China

**Keywords:** vector flow mapping, stress echocardiography, nonobstructive coronary artery disease, left ventricular flow field, treadmill exercise

## Abstract

**Purpose:**

Vector flow mapping and treadmill exercise stress echocardiography were used to evaluate and explore changes in the left ventricular (LV) flow field of patients with nonobstructive coronary artery disease.

**Methods:**

Overall, 34 patients with nonobstructive (<50%) left anterior descending coronary artery stenosis (case group) and 36 patients with no coronary artery stenosis (control group) were included. Apical four-, three-, and two-chamber echocardiographic images were collected at rest and during early recovery from treadmill exercise. LV flow field, vortex area, and circulation (cir) changes were recorded in different phases: isovolumetric systole (S1), rapid ejection (S2), slow ejection (S3), isovolumetric diastole (D1), rapid filling (D2), slow filling (D3), and atrial systole (D4). Intra- and inter-group differences were compared before and after exercise loading.

**Results:**

The control and case groups demonstrated regular trends of eddy current formation and dissipation at rest and under stress. Compared with the control group, the case group had irregular streamline distributions. Abnormal vortices formed in the S1 and D3 apical segments and D1 left ventricular middle segment in the resting group. Compared with the control group, the resting group had decreased left ventricular S1 vortex areas and increased S3 vortex areas. The post-stress D1 and D3 vortex areas and D1 and D2 cir increased. Compared with at rest, after stress, the control group had decreased S1, S3, D2, and D3 vortex areas; increased S2, D1, D3, and D4 cir; and decreased D2 cir. After stress, the case group had decreased S3 and D2 vortex areas, increased D1 vortex areas, and increased S2, D1, D3, and D4 cir (*P* all < 0.001). Logistic regression and ROC curve analyses show that increased D1 vortex area after stress is an independent risk factor for stenosis in nonobstructive stenosis of coronary arteries (OR: 1.007, 95% CI: 1.005–1.010, *P* < 0.05). A D1 vortex area cutoff value of 82.26 had an AUC, sensitivity, and specificity of 0.67, 0.655, and 0.726, respectively.

**Conclusion:**

The resting left ventricular flow field changed in patients with nonobstructive left anterior descending coronary artery stenosis. Both groups had more disordered left ventricular blood flow after stress. The increased D1 vortex area after stress is an independent risk factor for mild coronary stenosis and may contribute to the assessment of nonobstructive coronary stenosis. VFM combined with treadmill stress is useful in evaluating left ventricular flow field changes in patients with nonobstructive coronary artery disease, which is valuable in the early evaluation of coronary heart disease.

## 1 Introduction

Cardiovascular disease is the leading cause of death worldwide, accounting for more than 40% of deaths in China (1), where coronary artery disease is the leading cause of death (2). Coronary heart disease, i.e., coronary atherosclerotic heart disease (CAD), is a group of heart conditions caused by coronary artery atherosclerosis, which results in coronary artery lumen stenosis or occlusion, myocardial ischemia, hypoxia, or necrosis (3). In recent years, several large-sample studies have found that patients suspected of having CAD have a degree of coronary artery stenosis of less than 50%. These patients usually have various cardiovascular risk factors and are more likely to experience cardiovascular events than those without obvious coronary artery stenosis (4). Other studies have shown that adverse cardiac events, such as myocardial infarction and sudden cardiac death, mostly occur during coronary atherosclerotic plaque rupture or erosion, and most of these events are caused by non-severe coronary artery stenosis (5). The prognosis of patients with nonobstructive coronary artery disease (<50%) is worse than that of patients with moderate-to-severe coronary artery stenosis (>50%) (6). Therefore, the early evaluation of cardiac function in these patients is of great significance for improving their prognosis.

Stress echocardiography has become a reliable and economical method for diagnosing suspected or known CAD and risk stratification (7), and exercise stress echocardiography is the first choice for patients capable of exercise (8). Treadmill exercise stress echocardiography (TESE), a combination of echocardiography and treadmill exercise electrocardiography, is an effective and easy noninvasive examination method for determining CAD diagnosis and prognosis from the perspectives of cardiac structure, function, and electrophysiology. Exercise stress echocardiography can be used to determine the coronary flow velocity reserve of the left anterior descending branch and provide a comprehensive assessment of the epicardial coronary artery stenosis and microcirculation. Risk stratification is important for patients with coronary artery disease and heart failure (9). Studies have confirmed the prognostic role of stress echocardiography in patients with suspected coronary heart disease (10, 11). Blood flow vector imaging (VFM) is a new technique based on color Doppler ultrasonography for evaluating the hemodynamics of the cardiovascular system. VFM can display local blood flow velocity vectors in the cardiac cavity as vector graphs and streamline diagrams for qualitative and quantitative visual evaluations of the blood flow field to clarify the intracardiac hemodynamic characteristics (12, 13).

Myocardial ischemia caused by coronary artery stenosis can cause abnormal myocardial metabolism, affect myocardial contraction and relaxation, change the coordination of ventricular wall movement, increase blood flow resistance, and cause intracardiac hemodynamic changes. However, few studies have examined left ventricular flow field changes in patients with nonobstructive coronary artery disease, and research on left ventricular flow field changes in these patients after exercise stress is lacking. In this study, VFM and TESE were used to evaluate the left ventricular flow field of patients with nonobstructive coronary artery disease in order to further clarify the hemodynamic mechanism of left ventricular dysfunction and explore the value of VFM in these patients.

## 2 Study population and methods

### 2.1 Study population

Patients with suspected CAD and chest pain who were treated at Sichuan Provincial People's Hospital from July 2018 to April 2020 were selected. The inclusion criteria were as follows: (1) patients with nonobstructive stenosis of the left anterior descending coronary artery (<50%) on coronary angiography (CAG) or dual-source computed tomography (CT) coronary angiography (DSCTA) and (2) patients who underwent TESE within one week before CAG or DSCTA. The exclusion criteria were as follows: (1) age > 75 years; (2) severe hypertension (≥200/110 mmHg); (3) history of ischemic heart disease; (4) left ventricular ejection fraction <50%; (5) valvular heart disease with any degree of aortic regurgitation and other moderate lesions; (6) resting electrocardiogram (ECG) indicating left bundle branch block or other obvious arrhythmia; (7) cardiomyopathy or congenital heart disease; (8) acute or chronic respiratory disease; (9) hyperthyroidism and anemia or other diseases with a high hemodynamic state; and (10) poor ultrasonic imaging quality, inability to exercise, or uncooperative with the examination.

Based on the inclusion and exclusion criteria, 34 patients with nonobstructive stenosis of the left anterior descending coronary artery (case group) and 36 patients with no obvious coronary artery stenosis who underwent CAG or DSCTA during the same period (control group) were finally included.

This study was approved by the Ethics Committee, and all patients signed an informed consent form prior to the experiment.

### 2.2 Instrumentation and equipment

The Aloka ProSound F75 ultrasonic diagnostic instrument (Hitachi Company, Japan) and UST-52105 probe were used. The probe had a frequency range of 1.8–5 MHz. The Mortara was used with the exercise treadmill, and the instruments were the XScribe-Exercise ECG plate system and SunTech Tango synchronous dynamic sphygmomanometer.

### 2.3 Methods

Simultaneous complete dynamic images and blood pressure data before and after TESE were collected from patients in the case and control groups. The images were imported into the workstation in DICOM format. At rest, the thicknesses of the left ventricular posterior wall (LVPW) and interventricular septum (IVS) and the anterior and posterior diameters of the left atrium (LAD) were obtained from the parasternal left ventricular long-axis view. The early diastolic mitral flow velocity (E), late diastolic mitral flow velocity (A), and early diastolic velocity of the mitral lateral wall and septal annulus (e′) were obtained from the apical four-chamber (AP4C) view; the average velocity was calculated as the final result. In the apical three-chamber (AP3C) view, simultaneous sampling with bispectral Doppler (PW/PW) was used to adjust the baseline position of the mitral and aortic forward flow spectra, and the left ventricular Tei index was determined.

The left ventricular end-diastolic volume (EDV) and end-systolic volume (ESV) were measured using the biplane Simpson's method before and after exercise, and the ejection fraction (EF) and cardiac output (CO) were calculated in the AP4C and apical two-chamber (AP2C) views.

**Figure 1:**
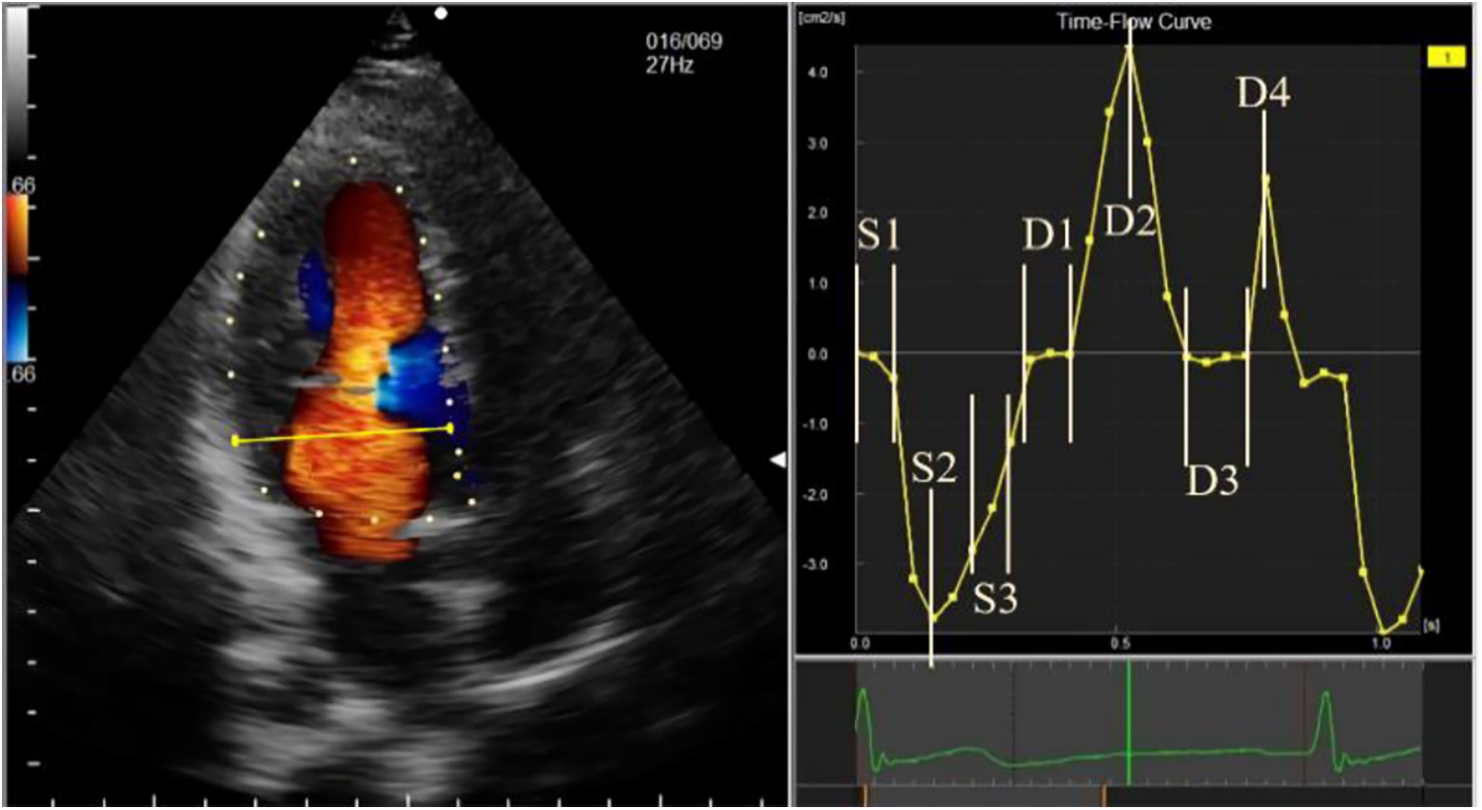
Schematic diagram of time-flow curve phase sampling. The phases are represented, as follows: S1, isovolumetric contraction; S2, rapid ejection; S3, slow ejection; D1, isovolumetric relaxation; D2, rapid filling; D3, slow filling; and D4, atrial systole.

The stored image was imported into the workstation in DICOM format, and a complete R-R cardiac cycle with full blood flow signals was selected to manually draw the endocardium. After the drawing was completed and successfully tracked, unsatisfactory endocardial boundaries were adjusted frame-by-frame. For images with blood flow signal aliasing, auto-aliasing was selected at the workstation to process the aliased blood flow signals, and unsatisfactory image frames were manually adjusted. The time-flow curve (TFC) was obtained from 2 cm on the mitral valve. Combining the TFC, ECG, and valve opening and closing, the cardiac cycle was divided into seven phases. The systolic phase included isovolumetric contraction (S1), rapid ejection (S2), and slow ejection (S3). The diastolic phases included isovolumetric relaxation (D1), rapid filling (D2), slow filling (D3), and atrial contraction (D4) (Figure 1). The vortex parameters were recorded in the frame displaying the most evident changes in different phases, including the vortex area and circulation (cir) in the left ventricular cavity. For phases with multiple vortices, the sum of the vortex area and the absolute value of cir was obtained when no vortices overlapped, and the area of the larger vortex and the absolute value of cir were obtained when the vortices overlapped. All vortex areas and cir used the average values of the AP4C, AP3C, and AP2C views measured simultaneously.

### 2.4 Statistical analysis

SPSS26.0 statistical software was used for the analysis. Counting data are expressed as frequency and percentage, and rates or constituent ratios between two groups were compared using the four-fold *χ*^2^ table. Measures are expressed as mean ± standard deviation if they conformed to a normal distribution and as median (upper and lower quartiles) if they did not. For normally distributed data, comparisons between the case and control groups before and after stress were performed using the independent samples *t*-test or *t*′ test, if the variance was not homogeneous; comparisons within groups before and after stress were performed using the paired samples *t*-test. For non-normally distributed data, the Mann-Whitney *U* test was used for between-group comparisons before and after stress, and the Wilcoxon paired rank-sum test was used for within-group comparisons before and after stress. Repeatability tests were performed using the intraclass correlation coefficient (ICC). Two-sided *P*-values < 0.05 were considered statistically significant.

### 2.5 Repeatability test

After 1 week, the ultrasound images from 10 cases were randomly selected from all subjects for repeatability testing. The original observer and another observer with similar experience arbitrarily chose a momentary phase and remeasured the parameters to assess the intra- and inter-observer repeatability of the measurements.

## 3 Results

### 3.1 Baseline characteristics

The sex, age, body mass index (BMI), body surface area (BSA), abdominal circumference, and number of patients who smoked, drank, had hyperglycemia, had hypertension, had hyperlipidemia, used beta-blockers, used nitrate medications, used calcium channel blockers (CCB), used statins, and used angiotensin receptor blockers (ARB) were not significantly different between the control and case groups (*P* > 0.05) (Table 1).

**Table 1:** Comparison of baseline characteristics between the two groups.

Parameters	Control group (*n* = 36)	Case group (*n* = 34)	*χ^2^/t*	*P*-value
Sex (male, %)	17 (47.22%)	15 (44.12%)	*χ^2^*=0.068	0.794
Age (y)	58.69 ± 10.26	58.94 ± 7.10	*t *= −0.118	0.907
BMI (kg/m^2^)	24.13 ± 3.00	24.48 ± 2.73	*t *= −0.511	0.611
BSA (m^2^)	1.71 ± 0.19	1.69 ± 0.17	*t *= 0.316	0.754
Abdominal circumference (cm)	86.19 ± 8.23	87.50 ± 7.73	*t *= −0.684	0.497
Smokers (%)	5 (13.89%)	10 (29.41%)	*χ^2 ^*= 2.502	0.114
Drinkers (%)	7 (19.44%)	9 (26.47%)	*χ^2 ^*= 0.49	0.484
Hyperglycemia (%)	4 (11.11%)	8 (23.53%)	*χ^2 ^*= 1.898	0.213
Hypertension (%)	16 (44.44%)	15 (44.12%)	*χ^2 ^*= 0.001	0.978
Hyperlipidemia (%)	12 (33.33%)	17 (50.00%)	*χ^2 ^*= 2.002	0.157
Beta-blockers (%)	7 (19.44%)	2 (5.88%)	*χ^2 ^*= 0.152	0.089
Nitrates (%)	2 (5.56%)	4 (11.76%)	*χ^2 ^*= 0.422	0.309
CCBs (%)	1 (2.78%)	0 (0.00%)	*χ^2 ^*= 1.000	0.514
Statins (%)	3 (8.33%)	8 (23.52%)	*χ^2 ^*= 3.049	0.081
ARBs (%)	6 (16.67%)	2 (5.88%)	*χ^2 ^*= 0.261	0.149

Values in the table are expressed as mean ± standard deviation or frequency (%). *t*- and *χ^2^*-values indicate *t*- and*χ^2^*-tests, respectively.

BMI, body mass index; BSA, body surface area; CCBs, calcium channel blockers; ARBs, angiotensin receptor blockers.

### 3.2 Serological indicators

The glucose (Glu), total cholesterol (TC), triglyceride (TG), low-density lipoprotein cholesterol (LDL-C), high-density lipoprotein cholesterol (HDL-C), hemoglobin (HGB), and hematocrit (HCT) levels and red blood cell counts (RBC) between the case and control groups were not significantly different (*P* > 0.05) (Table 2).

**Table 2:** Comparison of serologic indices between the two groups.

Parameters	Control group (*n* = 36)	Case group (*n* = 34)	*T*	*P*-value
Glu (mmol/L)	5.39 ± 1.00	5.21 ± 0.73	0.897	0.373
TC (mmol/L)	4.43 ± 0.80	4.07 ± 1.04	1.636	0.106
TG (mmol/L)	1.51 ± 0.87	1.53 ± 1.20	−0.070	0.944
LDL-C (mmol/L)	2.44 ± 0.68	2.24 ± 0.87	1.107	0.272
HDL-C (mmol/L)	1.32 ± 0.30	1.22 ± 0.42	1.218	0.228
RBC (10^12^/L)	4.54 ± 0.59	4.37 ± 0.41	1.389	0.170
HGB (g/L)	134.12 ± 15.51	134.22 ± 11.54	−0.029	0.977
HCT (%)	40.53 ± 3.93	40.25 ± 3.67	0.298	0.767

Values are expressed as mean ± standard deviation.

Glu, glucose; TC, total cholesterol; TG, triglyceride; LDL-C, low-density lipoprotein cholesterol; HDL-C, high-density lipoprotein cholesterol; RBC, red blood cell count; HGB, hemoglobin; HCT, hematocrit.

### 3.3 Routine echocardiographic indices

The diastolic LVPW, diastolic IVS, Tei index, LAD, E, A, E/A, e′, E/e, and EF were not significantly different between the case and control groups (*P* > 0.05) (Table 3).

**Table 3:** Comparison of conventional echocardiographic measurements between the two groups.

Parameters	Control group (*n* = 36)	Case group (*n* = 34)	*t/Z*	*P*-value
LVPW (mm)	7.75 ± 1.07	7.21 ± 1.37	*t *= 1.369	0.178
IVS (mm)	9.19 ± 1.76	8.38 ± 1.40	*t *= 1.691	0.098
Tei index	0.32 ± 0.07	0.29 ± 0.07	*t *= 1.008	0.322
LAD (mm)	32.60 ± 3.92	32.21 ± 3.55	*t *= 0.336	0.739
E (m/s)	0.63 ± 0.11	0.6 ± 0.14	*t *= 0.762	0.450
A (m/s)	0.13 ± 0.03	0.14 ± 0.03	*t *= −0.454	0.652
E/A	0.93 ± 0.20	0.86 ± 0.25	*t *= 0.983	0.331
e′ (m/s)	0.10 ± 0.01	0.09 ± 0.02	*t *= 1.645	0.107
E/e	6.56 ± 1.13	6.93 ± 1.56	*t *= −0.837	0.407
EF (%)	58.23 (57.23–60.40)Δ	60.00 (56.85–62.50)Δ	*Z *= −0.877	0.380

Values are expressed as mean ± standard deviation, frequency (%), or median (upper and lower quartiles). Δ indicates that the data do not follow a normal distribution. *t*-values indicate that the *t*-test was used. *Z*-values indicate that the rank-sum test was used.

LVPW, posterior wall of the left ventricle; IVS, interventricular septum; LA, left atrium diameter; E, early diastolic transmitral flow velocity; A, late diastolic transmitral flow velocity; e′, early diastolic velocity of the mitral annulus; EF, ejection fraction.

### 3.4 Echocardiographic indicators of exercise stress

At rest, the basal HR, HR at the time of image acquisition, resting systolic blood pressure (SBP), diastolic blood pressure (DBP), peak SBP, DBP, number of cases with wall motion score index (WMSI) > 1, left ventricular end-diastolic volume (EDV), end-systolic volume (ESV), EF, and cardiac output (CO) were not significantly different between the two groups (*P* > 0.05).

After stress, the peak HR, peak SBP, peak DBP, HR at the time of image acquisition, DBP at the time of image acquisition, SBP at the time of image acquisition, number of patients with WMSI > 1, EDV, ESV, EF, and CO were not significantly different between the two groups (*P* > 0.05).

The metabolic equivalents (METs) were not significantly different between the two groups (*P* > 0.05) (Table 4).

**Table 4:** Comparison of stress echocardiographic indices between the two groups.

Parameters	Control group (*n* = 36)	Case group (*n* = 34)	*t/Z*	*P*-value
Rest				
HR (*t*/min)	75.27 ± 9.88	80.41 ± 12.27	*t *= −1.832	0.072
HR at image acquisition (t/min)	69.12 ± 7.80	67.66 ± 10.56	*t *= 0.642	0.523
SBP (mmHg)	134.06 ± 12.82	130.76 ± 16.9	*t *= 0.894	0.374
DBP (mmHg)	78.82 ± 7.72	75.55 ± 11.09	*t *= 1.392	0.169
WMSI > 1 (%)	0 (0)^▴^	1 (2.94%)^▴^		0.486
EDV (ml)	75.44 ± 20.88	73.55 ± 17.83	*t *= 0.320	0.751
ESV (ml)	31.06 ± 7.87	29.24 ± 7.94	*t *= 0.739	0.464
EF (%)	58.23 (57.23–60.40)Δ	60.0 (56.85∼62.50)Δ	*Z *= −0.877	0.380
CO (L/min)	2.90 ± 0.77	3.03 ± 0.77	*t *= −0.549	0.586
Stress				
Peak HR (*t*/min)	159.5 (145.25–173)Δ	156 (144–164.5)Δ	*Z *= −1.369	0.171
Peak SBP (mmHg)	184.49 ± 20.69	178.94 ± 20.46	*t *= 1.119	0.267
Peak DBP (mmHg)	77.54 ± 11.76	75.91 ± 11.82	*t *= 0.575	0.567
WMSI > 1 (%)	1 (2.78%)^▴^	2 (5.88%)^▴^		0.609
HR at image acquisition (*t*/min)	90.16 ± 11.62	93.53 ± 10.57	*t *= −1.216	0.229
SBP at image acquisition (mmHg)	131.65 ± 12.42	129.00 ± 15.41	*t *= 0.678	0.501
DBP at image acquisition (mmHg)	66.23 ± 10.52	65.88 ± 10.64	*t *= 0.118	0.906
EDV (ml)	72.87 ± 23.06	68.57 ± 15.18	*t *= 0.750	0.457
ESV (ml)	21.33 ± 5.86	20.4 ± 5.14	*t *= 0.548	0.587
EF (%)	75.34 ± 3.60	75.06 ± 4.73	*t *= 0.202	0.841
CO (L/min)	4.72 ± 1.76	4.44 ± 1.13	*t *= 0.654	0.516
METs	9.30 (7.175–10.53)Δ	9.30 (6.50–10.95)Δ	*Z *= −0.476	0.634

Values are expressed as mean ± standard deviation, frequency (%), or median (upper and lower quartiles). Δ indicates that the data do not follow a normal distribution. *t*-values indicate that the *t*-test was used. *Z*-values indicate that the rank-sum test was used. *χ*2-values indicate that the *χ*2 test was used. ▴ indicates that the *χ*2-test was used with the Fisher's exact test.

HR, heart rate; SBP, systolic blood pressure; DBP, diastolic blood pressure; RWMA, ventricular wall motion integral index; EDV, end-diastolic volume; ESV, end-systolic volume; EF, ejection fraction; CO, cardiac output; METs, metabolic equivalent.

### 3.5 Qualitative observations of left ventricular VFM in the control group

In the resting state, the left ventricular basal segment at S1 in the control group formed a clockwise vortex, which lasted until the end of systole. The lateral velocity vectors of the left ventricular outflow tract at S2 and S3 were higher than that at S1, and the streamlines pointed from the apex to the left ventricular outflow tract. The counterclockwise vortex was formed in 25% of the patients during D1 and disappeared after mitral valve opening. In the rest of the patients, the streamlines and velocity vectors were gentle and pointed to the apex of the heart. The blood flow at D2 formed a vortex behind the mitral valve, and the streamlines were obliquely distributed to the apex along the direction of the mitral valve. The counterclockwise vortex at the back of the anterior lobe of the mitral valve extended into D3 and gently changed in the direction of the D3 velocity vector. With the inflow at D4, the posterior part of the mitral valve reformed a vortex, and the streamlines pointed to the apical area.

**Figure 2:**
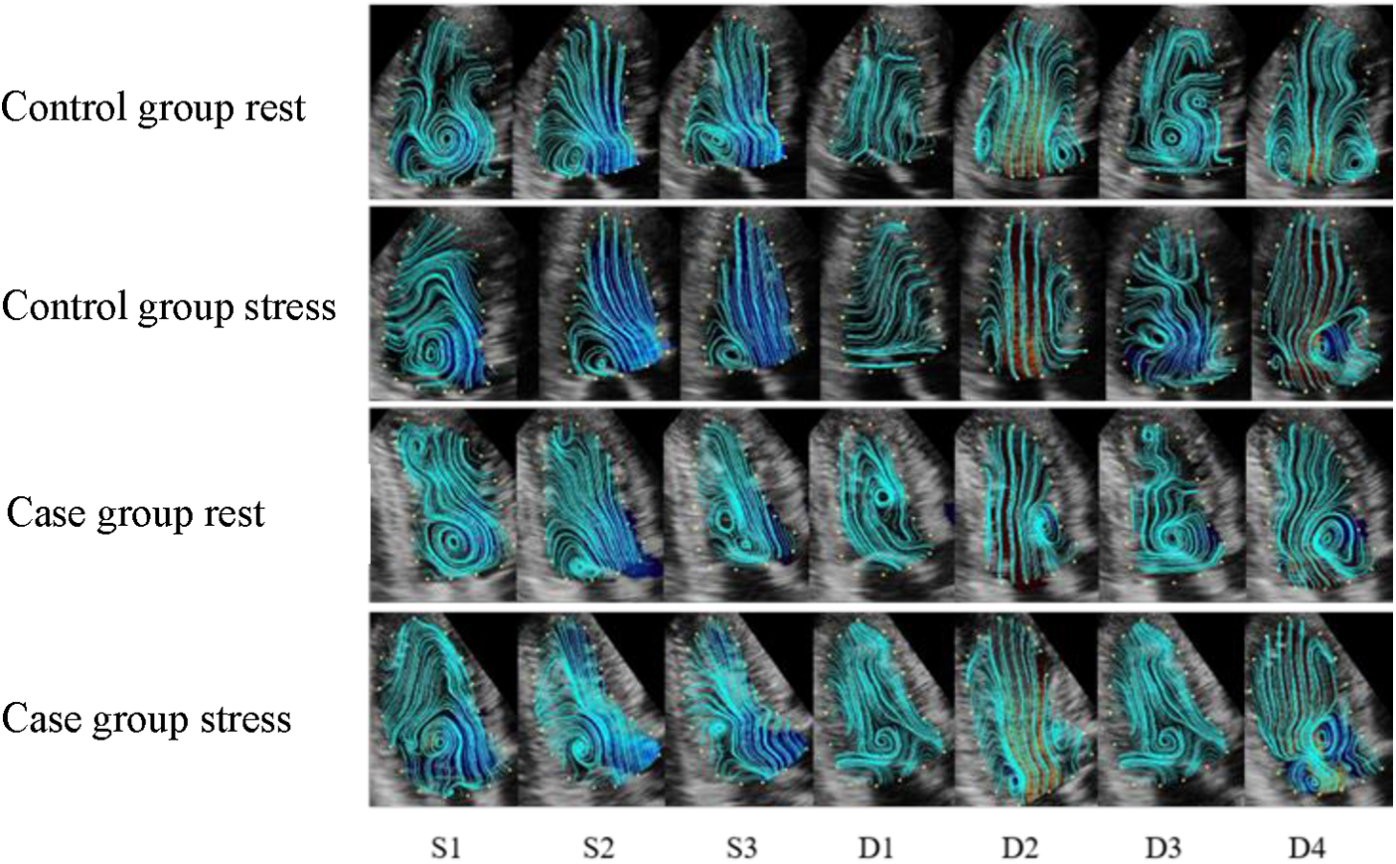
The streamline distribution of the left ventricle in the control and case groups. (S1) isovolumetric contraction period; (S2) rapid ejection period; (S3) slow ejection period; (D1) isovolumetric diastolic period; (D2) rapid filling period; (D3) slow filling period; (D4) atrial systole. The blue curve indicates the streamline distribution in the cardiac cavity.

**Figure 3:**
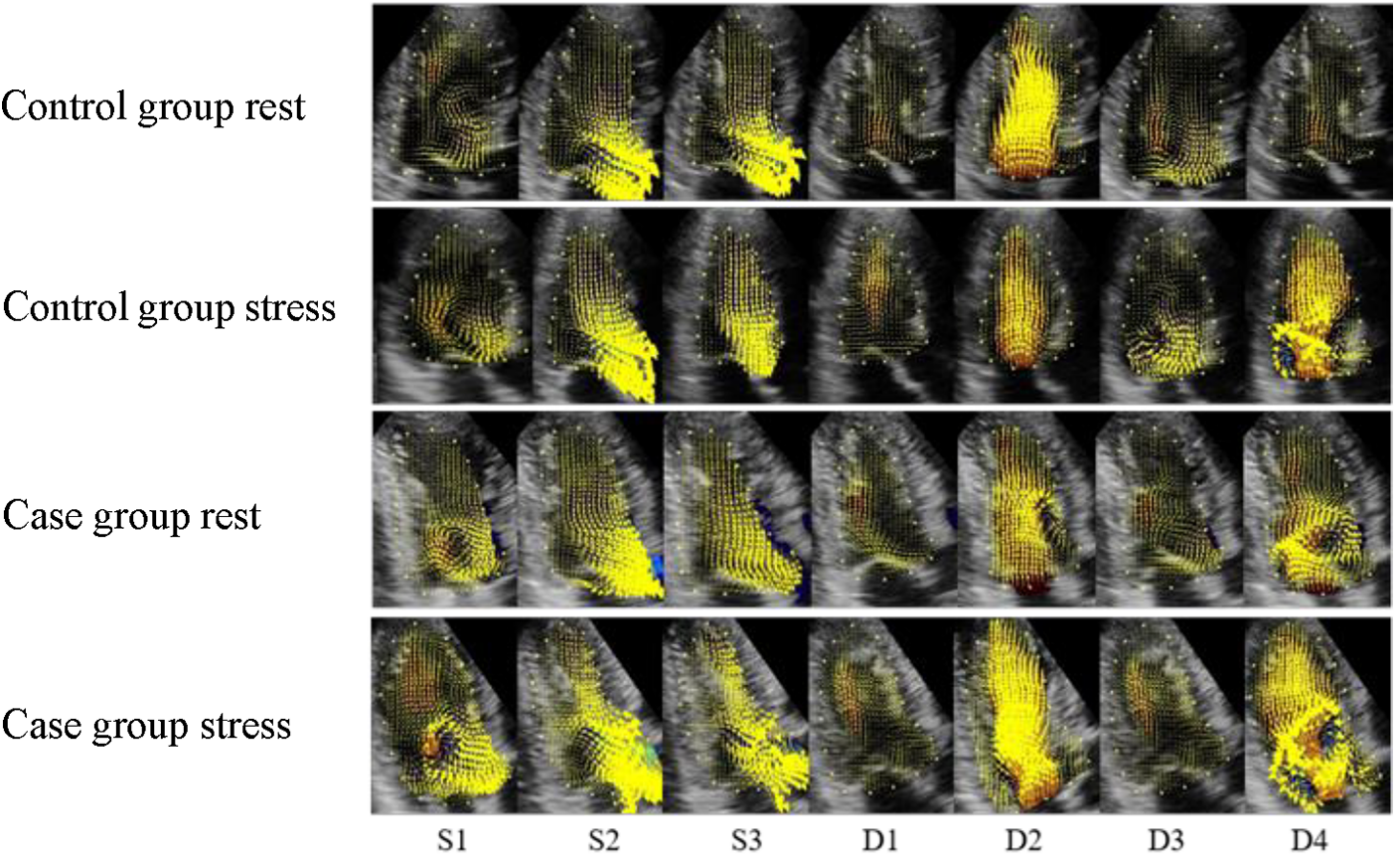
Velocity vector distribution maps of the control and case groups. (S1) isovolumetric contraction period; (S2) rapid ejection period; (S3) slow ejection period; (D1) isovolumetric diastolic period; (D2) rapid filling period; (D3) slow filling period; (D4) atrial systole. The yellow arrow represents the speed vectors, and the length of the arrows represents the size.

The elimination of the vortex after stress was consistent with that before stress. The vortex flow lines behind the mitral valve at S1 were disordered, and the direction of the velocity vectors changed noticeably. Thee velocity vector distribution on the side of the left ventricular outflow tract at S2 and S3 increased, and the flow lines from the apex to the left ventricular outflow tract were significantly greater than those before stress. In 33% of the patients, a counterclockwise vortex formed at D1, the streamline and velocity vector arrangement was slightly disordered compared with that before stress, and the velocity vector distribution in the apical segment increased. The D2 streamlines pointed from the mitral valve to the apex, and the velocity vectors at the posterior vortex of the mitral valve increased. The vortex duration in the D3 basal segment was shorter than that before stress, and the disorder of the streamlines and velocity vectors in the cardiac cavity was increased. At D4, the vortex behind the mitral valve was less regular and the arrangement of the streamlines and velocity vectors in the cardiac cavity was more disordered than those before stress (Figures 2, 3).

### 3.6 Qualitative observations of left ventricular VFM in the case group

In the resting state, a clockwise vortex formed in the basal segment at S1, and a smaller clockwise vortex was observed in the apical segment in approximately 33% of the patients. The basal vortex that formed at S1 lasted until the end of systole, and the lateral velocity vector of the left ventricular outflow tract was increased at S2 and S3. In approximately 67% of patients, a counterclockwise vortex formed at D1 in the middle of the cardiac cavity, and the streamlines approximately ran from the basal segment to the apical part of the heart. Following D2, inflow through the mitral valve occurred, and the vortex was only observed behind the mitral valve. The velocity vectors were concentrated on one side of the left ventricular inflow tract, and the streamlines ran obliquely along the mitral valve, towards the apex. In more than 50% of patients, the counterclockwise vortex behind the anterior lobe of the mitral valve lasted until D3, when a counterclockwise vortex formed in the apical segment. The velocity vector was disordered compared with that in the control group. In D4, the vortex formed behind the mitral valve. The streamlines ran to the apical segment, and the velocity vectors were disordered.

The elimination of the vortex after stress was consistent with that before stress. The S1 basal segment formed a clockwise vortex, and the velocity along the basal segment led to the left ventricular outflow tract. The S2 and S3 blood flow vectors on the side of the outflow tract were faster than those before stress, and the distribution was more disordered. Approximately 75% of patients formed a counterclockwise vortex at D1. The distribution of the velocity vectors in the apical segment was increased, and the streamlines were more disordered, compared with those before stress. The distribution of the velocity vectors at D2 was denser than that before stress. The duration of the vortex in the basal segment at D3 was shorter than that before stress. The vortex behind the mitral valve at D4 was irregularly shaped, and the streamlines and velocity vectors in cardiac cavity were more disordered than those before stress (Figures 2, 3).

### 3.7 Comparison of vortex areas between control and case groups

At rest, the S1 vortex area of the case group was significantly smaller (*P* < 0.05) and the S3 vortex area was significantly larger (*P* < 0.05) than those of the control group. The vortex areas of the other phases were not significantly different (*P* > 0.05) (Table 5).

**Table 5:** Comparison of resting state vortex areas in the control and case groups (mm^2^).

Parameters	Control group (*n* = 36)	Case group (*n* = 34)	*Z*	*P*-value
S1	424.86 (295.51–496.79)Δ	361.36 (185.02–544.29)Δ	−2.007	0.045*
S2	176.31 (90.99–232.23)Δ	190.73 (89.83–279.25)Δ	−0.315	0.753
S3	109.59 (0–192.17)Δ	141.28 (35.14–231.70)Δ	−2.087	0.037*
D1	28.63 (0–95.33)Δ	19.42 (0–155.00)Δ	−0.368	0.713
D2	168.28 (70.05–248.42)Δ	174.46 (82.72–337.81)Δ	−1.419	0.156
D3	286.50 (116.57–462.07)Δ	258.99 (93.55–386.91)Δ	−0.124	0.902
D4	296.43 (176.87–424.88)Δ	266.27 (210.86–431.20)Δ	−0.178	0.858

* Indicates that the comparison is statistically significant (*P* < 0.05 or <0.001). Δ indicates that data are not normally distributed, and the Mann-Whitney *U* test was used. S1, isovolumetric systole; S2, rapid ejection period; S3, slowed ejection phase; D1, isovolumetric diastole; D2, rapid filling phase; D3, slowed filling phase; D4, atrial systole.

Under stress, the D1 and D3 vortex areas of the case group were significantly larger after stress, compared with those of the control group, (*P* < 0.001). The vortex areas of the other phases were not significantly different (*P* > 0.05) (Table 6).

**Table 6:** Comparison of post-stress vortex areas of the control and case groups (mm^2^).

Parameters	Control group (*n* = 36)	Case group (*n* = 34)	*Z*	*P*-value
S1	351.95 (192.51–438.69)Δ	369.52 (256.41–537.00)Δ	−1.096	0.273
S2	185.33 (71.95–233.31)Δ	188.09 (93.43–236.61)Δ	−0.100	0.920
S3	25.40 (0–114.10)Δ	87.38 (0–146.00)Δ	−1.381	0.167
D1	27.91 (0–88.29)Δ	97.04 (26.31–167.99)Δ	−5.589	<0.001*
D2	99.39 (41.32–162.25)Δ	126.24 (49.34–159.69)Δ	−1.859	0.063
D3	228.06 (116.76–270.49)Δ	261.68 (228.19–335.09)Δ	−4.198	<0.001*
D4	280.05 (218.98–339.41)Δ	283.85 (152.75–358.94)Δ	−0.236	0.814

* Indicates that the comparison is statistically significant (*P* < 0.05 or <0.001). Δ indicates that the data are not normally distributed, and the Mann-Whitney *U* test was used; S1, isovolumetric systole; S2, rapid ejection phase; S3, slowed ejection phase; D1, isovolumetric diastole; D2, rapid filling phase; D3, slowed filling phase; D4, atrial systole.

### 3.8 Comparison of circulation between control and case groups

At rest, the cir at all phases in the case and control groups were not significantly different (*P* > 0.05) (Table 7).

**Table 7:** Comparison of resting state circulation in the control and case groups (m^2^/s).

Parameters	Control group (*n* = 36)	Case group (*n* = 34)	*Z*	*P*-value
S1	0.0138 (0.011–0.0200)Δ	0.0142 (0.0032–0.0222)Δ	−0.785	0.433
S2	0.0057 (0.0039–0.0112)Δ	0.0061 (0.0035–0.0115)Δ	−0.177	0.859
S3	0.0030 (0–0.0064)Δ	0.0038 (0.0012–0.0056)Δ	−1.635	0.102
D1	0.0003 (0–0.0020)Δ	0.0002 (0–0.0085)Δ	−1.777	0.076
D2	0.0096 (0.0031–0.0196)Δ	0.0094 (0.0029–0.0184)Δ	−0.813	0.416
D3	0.0088 (0.0029–0.0172)Δ	0.0079 (0.0025–0.0163)Δ	−0.203	0.839
D4	0.0138 (0.0090–0.0204)Δ	0.0126 (0.0048–0.0235)Δ	−0.904	0.366

* Indicates that the comparison is statistically significant (*P* < 0.05 or 0.001). Δ indicates that the data are not normally distributed, and the Mann-Whitney *U* test was used. S1, isovolumetric systole; S2, rapid ejection phase; S3, slowed ejection phase; D1, isovolumetric diastole; D2, rapid filling phase; D3, slowed filling phase; D4, atrial systole.

With stress, the D1 and D2 cir in the case group were significantly greater than those in the control group (*P* < 0.001), whereas the cir in other phases were not significantly different (*P* > 0.05) (Table 8).

**Table 8:** Comparison of post-stress cir in the control and case groups (m^2^/s).

Parameters	Control group (*n* = 36)	Case group (*n* = 34)	*Z*	*P*-value
S1	0.0154 (0.0062–0.0223)Δ	0.0141 (0.0094–0.0237)Δ	−0.262	0.794
S2	0.0095 (0.0043–0.0161)Δ	0.0085 (0.0023–0.017)Δ	−0.743	0.458
S3	0.0018 (0–0.0062)Δ	0.0034 (0–0.0074)Δ	−1.018	0.309
D1	0.0011 (0–0.0035)Δ	0.0022 (0–0.0113)Δ	−4.00	<0.001*
D2	0.0066 (0.0025–0.0123)Δ	0.0078 (0.0018–0.0221)Δ	−2.313	0.021*
D3	0.0157 (0.0066–0.0294)Δ	0.0154 (0.0073–0.0309)Δ	−0.258	0.797
D4	0.0190 (0.0145–0.0350)Δ	0.0190 (0.0049–0.0291)Δ	−1.773	0.076

* Indicates that the comparison is statistically significant (*P* < 0.05 or <0.001). Δ indicates that the data are not normally distributed, and the Mann-Whitney *U* test was used. S1, isovolumetric systole; S2, rapid ejection phase; S3, slowed ejection phase; D1, isovolumetric diastole; D2, rapid filling phase; D3, slowed filling phase; D4, atrial systole.

### 3.9 Comparison of vortex area and cir before and after stress in the control group

Compared with those in the resting state, the S1, S3, D2, and D3 vortex areas significantly decreased after stress in the control group (*P* < 0.05); the vortex areas in the other phases were not significantly different (*P* > 0.05). Compared with those in the resting state, the S2, D1, D3, and D4 cir were significantly increased (*P* < 0.05) and D2 cir was significantly decreased (*P* < 0.05) after stress in the control group; the cir in other phases was not significantly different (*P* > 0.05) (Tables 9, 10).

**Table 9:** Comparison of vortex areas before and after stress in control group (mm^2^).

Parameters	Rest	Stress	*Z*	*P-*value
S1	424.86 (295.51–496.79)Δ	351.95 (192.51–438.69)Δ	−2.260	0.024*
S2	176.31 (90.99–232.23)Δ	185.33 (71.95–233.31)Δ	−0.320	0.749
S3	109.59 (0–192.17)Δ	25.40 (0–114.10)Δ	−2.508	0.012*
D1	28.63 (0–95.33)Δ	27.91 (0–88.29)Δ	−0.848	0.397
D2	168.28 (70.05–248.42)Δ	99.39 (41.32–162.25)Δ	−4.236	<0.001*
D3	286.50 (116.57–462.07)Δ	228.06 (116.76–270.49)Δ	−2.751	0.006*
D4	296.43 (176.87–424.88)Δ	280.05 (218.98–339.41)Δ	−0.298	0.766

* Indicates that the comparison is statistically significant (*P* < 0.05 or 0.001). Δ indicates that the data are not normally distributed, and the two paired-samples Wilcoxon rank-sum test was used. S1, isovolumetric systole; S2, rapid ejection; S3, slowed ejection; D1, isovolumetric diastole; D2, rapid filling period; D3, slow filling period; D4, atrial systole.

**Table 10:** Comparison of circulation before and after stress in the control group (m^2^/s).

Parameters	Rest	Stress	*Z*	*P-*value
S1	0.0138 (0.0110–0.0200)Δ	0.0154 (0.0062–0.0223)Δ	−0.265	0.791
S2	0.0057 (0.0039–0.0112)Δ	0.0095 (0.0043–0.0161)Δ	−4.532	<0.001*
S3	0.0030 (0–0.0064)Δ	0.0018 (0–0.0062)Δ	−0.121	0.903
D1	0.0003 (0–0.0020)Δ	0.0011 (0–0.0035)Δ	−2.066	0.039*
D2	0.0096 (0.0031–0.0196)Δ	0.0066 (0.0025–0.0123)Δ	−3.822	<0.001*
D3	0.0088 (0.0029–0.0172)Δ	0.0157 (0.0066–0.0294)Δ	−5.308	<0.001*
D4	0.0138 (0.009–0.0204)Δ	0.0190 (0.0145–0.0350)Δ	−5.391	<0.001*

* Indicates that the comparison is statistically significant (*P* < 0.05 or 0.001), Δ indicates that the data were not normally distributed, and the two paired-samples Wilcoxon rank-sum test was used. S1, isovolumetric systole; S2, rapid ejection; S3, slowed ejection; D1, isovolumetric diastole; D2, rapid filling period; D3, slow filling period; D4, atrial systole.

### 3.10 Comparison of vortex area and circulation before and after stress in the case group

Compared with those of the resting state, the S3 and D2 vortex areas were significantly decreased (*P* < 0.001) and the D1 vortex area was significantly increased (*P* < 0.001) after stress in the case group; the vortex areas in the other phases were not significantly different (*P* > 0.05). Compared with those of the resting state, the S2, D1, D3, and D4 cir were significantly increased after stress in the case group (*P* < 0.05); the cir in the other phases was not significantly different (*P* > 0.05) (Tables 11, 12).

**Table 11:** Comparison of vortex areas before and after stress in the case group (mm^2^).

Parameters	Rest	Stress	*Z*	*P-*value
S1	361.36 (185.02–544.29)Δ	369.52 (256.41–537.00)Δ	−0.635	0.525
S2	190.73 (89.83–279.25)Δ	188.09 (93.43–236.61)Δ	−0.724	0.469
S3	141.28 (35.14–231.70)Δ	87.38 (0–146.00)Δ	−4.041	<0.001*
D1	19.42 (0–155.00)Δ	97.04 (26.31–167.99)Δ	−3.923	<0.001*
D2	174.46 (82.72–337.81)Δ	126.24 (49.34–159.69)Δ	−5.051	<0.001*
D3	258.99 (93.55–386.91)Δ	261.68 (228.19–335.09)Δ	−0.870	0.384
D4	266.27 (210.86–431.20)Δ	283.85 (152.75–358.94)Δ	−0.041	0.967

* Indicates that the comparison is statistically significant (*P* < 0.05 or 0.001). Δ indicates that the data are not normally distributed, and the two paired-samples Wilcoxon rank-sum test was used. S1, isovolumetric systole; S2, rapid ejection; S3, slowed ejection; D1, isovolumetric diastole; D2, rapid filling period; D3, slow filling period; D4, atrial systole.

**Table 12:** Comparison of circulation before and after stress in the case group (m^2^/s).

Parameters	Rest	Stress	*Z*	*P-*value
S1	0.0142 (0.0032–0.0222)Δ	0.0141 (0.0094–0.0237)Δ	−1.543	0.123
S2	0.0061 (0.0035–0.0115)Δ	0.0085 (0.0023–0.0170)Δ	−3.438	0.001*
S3	0.0038 (0.0012–0.0056)Δ	0.0034 (0–0.0074)Δ	−0.489	0.625
D1	0.0002 (0–0.0085)Δ	0.0022 (0–0.0113)Δ	−2.898	0.004*
D2	0.0094 (0.0029–0.0184)Δ	0.0078 (0.0018–0.0221)Δ	−0.302	0.763
D3	0.0079 (0.0025–0.0163)Δ	0.0154 (0.0073–0.0309)Δ	−5.771	<0.001*
D4	0.0126 (0.0048–0.0235)Δ	0.0190 (0.0049–0.0291)Δ	−2.583	0.010*

* Indicates that the comparison is statistically significant (*P* < 0.05 or 0.001). Δ indicates that the data are not normally distributed, and the two paired-samples Wilcoxon rank-sum test was used. S1, isovolumetric systole; S2, rapid ejection; S3, slowed ejection; D1, isovolumetric diastole; D2, rapid filling period; D3, slow filling period; D4, atrial systole.

### 3.11 Logistic regression and ROC curve analysis results

**Figure 4:**
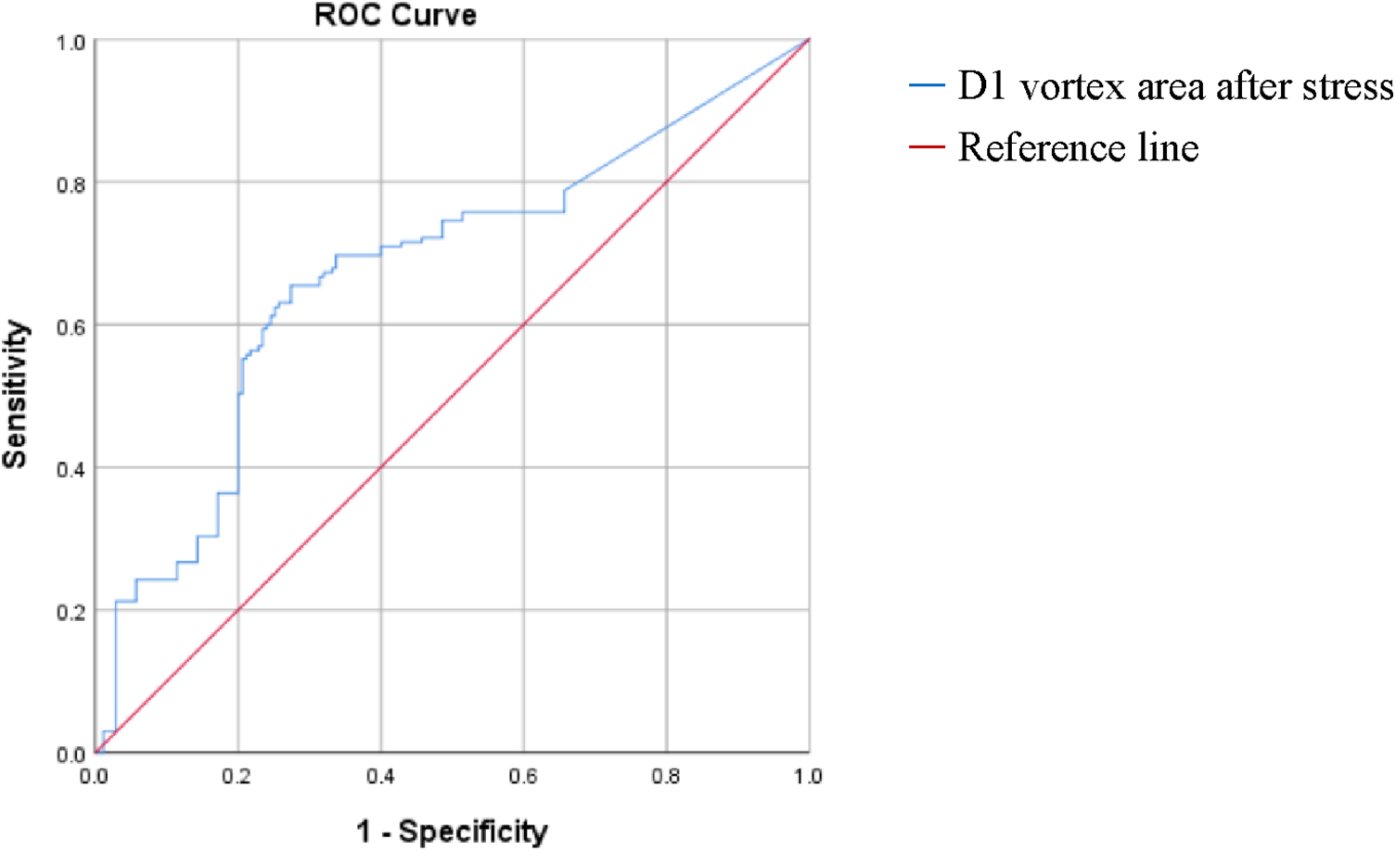
ROC curve analysis. D1 indicates the isovolumetric diastolic phase.

One-way binary logistic regression analyses of the vortex area and cir before and after stress were performed. The binary logistic regression equation was constructed by incorporating the D1 vortex area after stress (*P* < 0.20 and goodness-of-fit). The results revealed that increased D1 vortex area after stress was an independent risk factor for mild nonobstructive stenosis of the coronary arteries (OR: 1.007, 95% CI: 1.001 to 1.004, *P* < 0.05). The ROC curve analysis showed that using a post-stress D1 vortex area cutoff value of 7.23 resulted in an AUC of 0.65, a sensitivity of 0.636, and a specificity of 0.657 (Tables 13, 14, Figure 4).

**Table 13:** Logistic regression analysis.

Variant	*Β*	SE	Wald	*P*	OR (95% CI)
D1 vortex area after stress	0.007	0.001	26.707	<0.001*	1.007 (1.005–1.010)

* Indicates a statistically significant regression analysis (*P* < 0.05 or 0.001).

**Table 14:** Regression analysis of ROC curves.

Variant	AUC	95% CI	*P*	Truncation value	Sensitivity	Specificity
D1 vortex area after stress	0.67	0.615–0.732	<0.001*	82.26	0.655	0.726

* Denotes *P* < 0.05.

### 3.12 Repeatability test

Intra- and inter-observer repeatability tests were performed for the left ventricular vortex area and cir. The intra-observer ICC for the resting vortex area was 0.992 (*P* < 0.001), the inter-observer ICC was 0.978 (*P* < 0.001), the intra-observer ICC for cir was 0.994 (*P* < 0.001), and the inter-observer ICC was 0.935 (*P* < 0.001). The intra-observer ICC for the post-stress vortex area was 0.972 (*P* < 0.001), the inter-observer ICC was 0.966 (*P* < 0.001), the intra-observer ICC for cir was 0.874 (*P* < 0.001), and the inter-observer ICC was 0.807 (*P* < 0.05) (Table 15).

**Table 15:** Repeatability test.

	Intra-observer		Inter-observer	
	ICC	*P*	ICC	*P*
Rest				
Vortex area	0.992	<0.001*	0.978	<0.001*
cir	0.994	<0.001*	0.935	<0.001*
Stress				
Vortex area	0.972	<0.001*	0.966	<0.001*
cir	0.874	<0.001*	0.807	0.001*

* Indicates that the repeatability test is statistically significant (*P* < 0.05 or 0.001).

## 4 Discussion

VFM combined with two-dimensional speckle tracking generates a velocity vector field by post-processing the color Doppler velocity data obtained from conventional echocardiography and reflects hemodynamic changes in the cardiac cavity and vascular lumen under different physiological conditions. This is an effective method for visualizing the cardiac blood flow field (14). Intracardiac blood flow has two types of flow field states, laminar flow and eddy currents, in different phases of the cardiac cycle. VFM calculates the velocity vectors of blood flow in the cardiac cavity using a continuous equation, displays the intracardiac flow field through different imaging modes, overcomes the angle dependence of traditional imaging, allows qualitative and quantitative assessments of the intracardiac blood flow field, and distinguishes laminar flow and eddy currents (15). In the VFM vortex imaging mode, the position of the closed flow line of the annular vortex is the vortex, the area of the closed flow line is the vortex area, and the integral of the velocity component in the tangential direction of the closed flow line is the cir, which reflects the sum of the vortex rotation direction and intensity. The fluid velocity increases, and the cir increases (16). Friction occurs between the blood flowing in the cardiac cavity and the shear flow of the ventricular wall; the kinetic energy caused by the friction is converted into thermal energy, which is the energy loss. Eddy currents are an effective form of natural energy storage. Eddy currents may increase the shear stress at the ventricular wall, but they can maintain the blood flow stability and reduce energy loss (17). Previous studies confirmed that evaluating eddy current models has great clinical significance for better understanding the left ventricular function (18–21). The previous application of VFM in CAD studies shows that VFM can be used to quantitatively evaluate the changes of intracardiac hemodynamics in patients with CAD (22). With increasing degrees of coronary artery stenosis, changes in the left ventricular flow field are more obvious (23), and VFM can quantitatively evaluate the relative changes in the left ventricular pressure in patients with coronary heart disease. The relative decrease in the left ventricular pressure in patients with cardiac insufficiency is more obvious than that in patients with normal cardiac function and is closely related to the left ventricular diastolic function (24). VFM can also be used to study the intravalvular blood flow in patients with valvular disease or hypertrophic cardiomyopathy to evaluate the interaction between the cardiac blood flow and valvular motion (13–26).

In this study, changes in the left ventricular flow field in patients with nonobstructive coronary artery disease were evaluated using VFM combined with TESE. Changes in the left ventricular flow field were compared between patients with nonobstructive stenosis of the left anterior descending coronary artery and those with no obvious coronary artery stenosis. The study aimed to explore the difference in the left ventricular flow field between the two groups and compare the difference in the left ventricular vortex area and circulation in different phases to provide a basis for the evaluation of cardiac function in patients with nonobstructive coronary artery disease. In this study, abnormal blood flow patterns were formed in patients with nonobstructive coronary artery disease at rest, and the eddies in different phases were changed, as compared with those in the control group. After stress, disturbances in the flow field were more significant in patients with nonobstructive coronary artery disease. In the different phases, the vortex area in the case group was larger than that in the control group, especially at D1 and D3 (*P* < 0.05).

In this study, we found that in the resting state, the vortex area of the control group decreased at S1–S3, the maximum vortex area for the entire cardiac cycle occurred at S1, the minimum vortex area for the entire cardiac cycle occurred at D1, and the vortex area increased at D1–D4. In the control group, the trends of the cir and vortex area changes were nearly similar in different cardiac cycles. The cir showed an increasing trend at S1–S3, and the maximum cir for the entire cardiac cycle occurred at S1. The minimum cir occurred at D1, D1–D2, and D4; the cir increased at D3; and the cir at D3 was less than that at D2. This may be because the left ventricular pressure rises sharply at S1, no blood flows in or out of the left ventricle, a continuous and overall large eddy current occurs in the left ventricular cavity, the vortex area increases, and the cir increases. The significance not only lies in the transformation of the blood flow direction in the cardiac cavity but also in the effective energy transfer of the blood flow in preparation for the rapid ejection of blood (27); during S2–S3, the blood in the left ventricle flows to the aorta, the left ventricular cavity pressure decreases, the residual blood flow in the left ventricle decreases, the eddy current decreases, the vortex area decreases, and the corresponding cir also decreases. At D1, the left ventricular pressure decreases rapidly, the left ventricular volume and blood flow decreases to the lowest levels, the vortex area decreases, and the corresponding cir also decreases. During D2–D4, the left atrial blood flows into the left ventricle, the left ventricular blood flow gradually increases, and the vortex area gradually increases. At D2, the left atrial pressure is greater than that of the left ventricle, and the left atrial blood quickly flows into the left ventricle and forms an eddy current. At D3, the mitral valve is in a semi-closed state, and the left atrial blood flows slowly into the left ventricle. Because the cir is related to the fluid velocity, the cir at D3 is less than that at D2. At D4, the left atrium actively contracts, the left atrial blood quickly flows into the left ventricle, and the cir is greater than that at D3.

The trends of the vortex area and cir changes in the resting and control groups were the same. However, compared with those in the control group, the S1 apical vortex decreased and the S3 vortex area increased in some patients in the case group. Thus, for patients with nonobstructive coronary artery disease, the coordination of movement between the ischemic subendocardial myocardium and surrounding normal myocardium decreased due to repeated subendocardial ischemia in the ischemic segment of the left ventricle. Uncoordinated wall contractions increase the ineffective work of the heart and reduce the blood transmission efficiency. At S1, an effective pressure gradient to guide the blood flow fails to form. As a carrier that guides blood flow direction changes, the vortex area decreases. Under the decreased overall blood transfer efficiency, generating abnormal blood flow patterns further increases the subsequent blood flow pump resistance, slows the rate of decrease in intracardiac pressure compared with that in the control group, and increases the S3 residual blood flow. The intracardiac vortex area increases. The basal segment of left ventricle has more coronary artery branches, a more abundant blood supply than that in the middle and apical segments, and the least sensitivity to blood flow changes (28). The contractile effect of the heart depends on the torsion of the basal and apical segments in different directions, and the torsion of the apical segment is more evident than that of the basal segment (29). The ischemic subendocardial myocardial torsion decreases, the apical segment fails to form an effective pressure gradient distribution, and the guiding effect on blood flow is reduced, resulting in the formation of the S1 apical vortex. The diastolic blood flow state is more complex than that in the contractile phase. Left ventricular diastolic dysfunction is a sensitive early indicator of myocardial ischemia that lasts longer than systolic dysfunction (30). D1 is the initial stage of left ventricular filling. According to the myocardial band model of Guasp et al. (31), during systole, the basal ring contracts, the apical ring increases, the intracardiac area decreases, pressure increases, and blood flows out. At D1, the basal segment increases, the subendocardial myocardium lengthens longitudinally, the ventricular wall relaxes, and the degree of cardiac torsion gradually decreases, thus reducing the intraventricular pressure and preparing for blood flow filling at D2. In patients with nonobstructive coronary artery disease, the ventricular torsion motion was underbalanced, and eddy current formation in the middle segment was observable because the prolongation of the subendocardial myocardium and shortening of the subepicardial myocardium could not be completely matched at D1. At D3, the left ventricle actively fills via the blood flow through the low-pressure gradient between the atrium and ventricle, the anterior lobe of the mitral valve is in a semi-closed state, and a large counterclockwise eddy current forms in the left ventricle (32).

After stress, the trends of the vortex area and cir changes in the control group were the same, with a decreasing trend at S1–S3. The maximum vortex area and cir occurred at S1, while minimum occurred at D1. The vortex area and cir increased at D1–D4. Compared with those in the resting state, the vortex area decreased at S1, S3, D2 and D3 and increased at S2, D1, and D3; the D4 cir increased; and the D2 cir decreased after stress in the control group. This may be due to the increased heart rate, rapid left ventricular wall movement, and decreased cardiac chamber area after stress, especially at S1, S3, D2, and D3. The left ventricular pressure increased, forming a vortex with a smaller area; increased heart rate and a marked increase in blood flow velocity, predominantly S2, D1, D3, and D4, along with an increase in cir, the D2 time was shortened, the corresponding blood flow decreased, and the cir decreased during stress. After stress, the trends in vortex area and cir changes in the case group were the same as those in the control group. However, the vortex area, especially at D1 and D3, and the D1 and D2 cir in the case group were significantly larger than those in the control group. Compared with the resting state, the trends of vortex area and cir changes in the case group were almost the same as those in the control group, with decreased S3 and D2 vortex areas, an increased D1 vortex area, and increased S2, D1, D3, and D4 cir. It may be that after stress, the myocardial oxygen demand increases with the increased exercise volume, and patients with nonobstructive coronary artery disease experience further aggravation of the incoordination of the ventricular wall motion and more obvious segmental motion abnormalities. The subendocardial myocardial contractile function further decreases, aggravating the degree of uncoordinated torsion movement, decreasing the blood pumping efficiency, prolonging the cardiac ejection time, and increasing the blood flow. Therefore, the left ventricular vortex area in the case group was significantly larger than that in the control group, especially at D1 and D3. After stress, the left ventricular blood flow velocity and eddy current increased, especially at D1 and D2; therefore, the D1 and D2 cir were higher than those of the control group. Zhou et al. (33) showed that changes in the diastolic cir and vortex area reflect the abnormal filling of left ventricular diastolic blood flow. Therefore, cir and eddy current area changes can reflect cardiac blood flow changes. Logistic regression analysis suggests that an increased D1 vortex area after stress was an independent risk factor for mild nonobstructive stenosis of the coronary arteries. A D1 vortex area cutoff value of 82.26 had an AUC, sensitivity, and specificity of 0.67, 0.655, and 0.726, respectively, further confirming that the vortex area has a certain value in the evaluation of patients with nonobstructive coronary artery disease.

Patients with nonobstructive coronary artery disease may develop further at a later stage, resulting in localized myocardial disorders due to insufficient perfusion, abnormal myocardial segmental motion, and even heart failure, causing disturbances in cardiac chamber hemodynamics. VFM combined with exercise stress can intuitively show the change rule of blood flow field in the heart cavity, and can initially quantitatively analyze the change characteristics of the blood flow state in the left ventricular cavity at different stages of dynamic circulation, which provides a new method for the early diagnosis of non-obstructive coronary artery disease, and helps to improve the prognosis of the patients after the diagnosis is clear and timely treatment.

## 5 Study limitations

The sample size of this study was small, only the coronary artery anatomy was used to classify patients, and normal case controls were lacking. Thus, the myocardial function and microcirculation of the patients require further research. The blood flow pattern in the intracardiac cavity is three-dimensional, but the current VFM imaging only displays a two-dimensional plane. Therefore, improvements are required to accurately identify the intracardiac flow field. Due to a limitation of the ProSound F75 instrument, although the image frame rate was increased as much as possible after stress, the actual image frame rate was still low. The time phase selected in this study did not fully correspond to real time, and the actual parameters were underestimated.

## 6 Conclusion

The left ventricular flow field changes in patients with nonobstructive left anterior descending coronary artery stenosis at rest. The left ventricular blood flow in both groups is more disordered after stress, and increased D1 vortex area after stress is an independent risk factor for mild coronary stenosis and may contribute to the assessment of nonobstructive coronary stenosis. VFM combined with exercise stress can be used to further evaluate left ventricular flow field changes in patients nonobstructive coronary artery disease, which has value in the early evaluation of coronary heart disease.

## Data Availability

The original contributions presented in the study are included in the article/Supplementary Material, further inquiries can be directed to the corresponding author.
